# Effects of Genotype and Housing System on Rabbit Does’ Aggressive Behaviors and Injuries in Smallholding Conditions

**DOI:** 10.3390/ani13081357

**Published:** 2023-04-15

**Authors:** Ondřej Krunt, Lukáš Zita, Adam Kraus, Ágnes Moravcsíková, Martina Frühauf Kolářová, Luděk Bartoš

**Affiliations:** 1 Department of Animal Science, Faculty of Agrobiology, Food and Natural Resources, Czech University of Life Sciences Prague, Kamýcká 129, Suchdol, 165 00 Prague, Czech Republic; krunt@af.czu.cz (O.K.);; 2 Department of Ethology and Companion Animal Science, Faculty of Agrobiology, Food and Natural Resources, Czech University of Life Sciences Prague, Kamýcká 129, Suchdol, 165 00 Prague, Czech Republic; moravcsikova@af.czu.cz; 3 Institute of Animal Science, Přátelství 815, 104 00 Prague, Czech Republic; bartos@vuzv.cz; 4 Department of Veterinary Sciences, Faculty of Agrobiology, Food and Natural Resources, Czech University of Life Sciences Prague, Kamýcká 129, Suchdol, 165 00 Prague, Czech Republic; fruhauf_kolarova@af.czu.cz; 5 Department of Game Management and Wildlife Biology, Faculty of Forestry and Wood Sciences, Czech University of Life Sciences Prague, Kamýcká 129, Suchdol, 165 00 Prague, Czech Republic

**Keywords:** aggression suppression, behaviour, burrows, injury, kits, smallholders

## Abstract

**Simple Summary:**

The housing of rabbit does in groups is nowadays a subject of study in the scientific literature due to the public’s concerns about animals’ welfare. Group housing of rabbits brings more social contacts and more space for manifesting their species–specific behaviours. However, group living has the potential to be more natural as rabbits live in colonies in nature, but the opposite is true. Problems with aggression among rabbit does are common in this type of housing. In addition, there are direct aggressive attacks by does towards kits from other mothers and this leads to economic losses due to higher kit mortality. There are various efforts to solve aggression among females, but most of them work with the implications of these procedures in intensive breeding, where the purchase prices of technology are very expensive. Therefore, this study deals with the solution to the given issue at the level of small farms, where the attention of the scientific sphere has been minimal.

**Abstract:**

The objective of the study was to investigate the effects of housing (deep litter + concrete floor vs. deep litter + ground soil with the possibility to dig burrows), and genotype (Mecklenburg or Hyplus) on aggressive behaviour, social contacts, does’ and kits’ injuries, and progeny mortality. Twelve groups of six rabbit does (n = 72) were assigned to four treatments (two housing systems and two genotypes). Aggressive behaviour of does, number of injuries on does and kits, and postnatal kit mortality were recorded. The effects of housing and genotype were tested using multivariate GLMM Models. We found that the housing treatment in interaction with the genotype had a significant effect on aggressive behaviours in group housed does (*F*_3,12_ = 14.34, *p* = 0.0003), where the lowest incidence of aggression was in Mecklenburg does housed on ground soil. Reduced aggression was reflected in a lower number of injuries in does (*F*_3,68_ = 10.51, *p* < 0.0001), number of injuries in kits, and kit mortality (*F*_3,1_ = 4.59, *p* < 0.0001, *F*_3,54_ = 43.94, *p* < 0.0001). The results indicate that the proper combination of genotype and housing should be carefully considered for breeding to reduce aggression and injury in group housed does.

## 1. Introduction

European wild rabbits (*Oryctolagus cuniculus*) live in colonies with their specific hierarchy, which is achieved by territorial behaviour. Rabbit groups usually consist of bucks two–three), females (two–nine), and their offspring. The hierarchy between males and females manifests as linear. Females fight for breeding sites and males for territory and mates [1]. The dominant position among rabbits linearly changes in subsequent years according to their condition and is achieved by territorial and aggressive behaviour. Fights between the females are frequent at the beginning of breeding when strange animals are released together and gradually decrease after the social stabilisation of the group. Fighting is then rarer than in the beginning [2]. The trend of recent years is to bring the housing of rabbit does as close as possible to their natural behaviour (e.g., group housing). Regardless of the efforts of scientists to reduce aggressive behaviour between does kept on the farm, the aggressiveness of does towards each other is still a problem. Therefore, the females are usually single housed in a wire cage with their litter until weaning [3]. Aggression among rabbit does in group housing systems has been recently reported in several studies [4,5,6]. These studies investigated the influence of the group size, pen characteristics, or pen floor type on the behaviour and welfare of does. Typically, the aggression mainly took place during the first hour after grouping [4]. Scientific research has focused on two types of group housing systems (continuous and part-time) in recent years [7]. The authors summarised that continuous group housing resulted in a high number of aggressive events and injuries in does, which is incompatible with animal welfare. They see the potential in part-time group housing; however, the final procedure which could be applied to farms has not been developed yet. Szendrő et al. [8] reviewed the use of hiding places, which reduced the number of does with skin injuries and were shown to be promising in group-housed systems [9]. Although many studies have looked at the effect of the housing system, there are very few that take into account the effect of rabbit genotype; however, there were some indicia (more aggressive Dutch rabbits, compared to New Zealand ones) which revealed this possibility to be relevant [10].

There are nearly 161,000 registered backyard farms and 4500 commercial farms with 180 million rabbits for meat production in the EU [11]. Backyard farming is divided into three classes: small, medium, and large farms. The farmers usually follow family traditions in breeding animals, doing as their parents or grandparents did. Owners of medium and large backyard farms are usually interested in expanding their knowledge and improving the quality of animals’ lives as well as farm production. Intensive rabbit production is typical in the EU, particularly for countries such as Italy, France, Spain, and Hungary. On the other hand, small and medium farms are spread all around the world, predominating in South America and Asia. For these farms, scientific knowledge is essential [12]. Moreover, there are backyard farms, which have limited resources, and farming systems are on the “low input” level in Africa. The housing systems are composed of local materials, and rabbit consumption usually takes place in the farmer´s house or local market [13]. Typically, native, or coloured breeds are used in small or medium size farms for meat production. Rabbit does are bred there naturally when the doe is released into the buck´s cage for mating [12]. Based on the literature research, we designed an alternative housing system for group-housed does for smallholders. The rearing system was based on the possibility of making burrows (does are housed on the ground consisting of a layer of soil with deep littering manifesting their species-specific activities, e.g., digging). We have compared this housing system with deep litter housing (both with a male presence) with the concrete ground, where the does could not make burrows. Moreover, we used Hyplus genotype rabbits that are commercially used, and the original breed Mecklenburg checked rabbits for comparison.

We hypothesised that rabbit does with the possibility of digging burrows will show aggressive behaviour towards other females less often, will have fewer skin injuries, fewer kits with skin injuries, and lower kit mortality than does from deep litter housing. Furthermore, Mecklenburg checked rabbit does with the possibility of making burrows will show aggressive behaviour towards other females less often, will have fewer skin injuries and fewer kits with skin injuries, and have lower kit mortality than Hyplus females.

## 2. Materials and Methods

### 2.1. Animals and Housing

**Figure 1:**
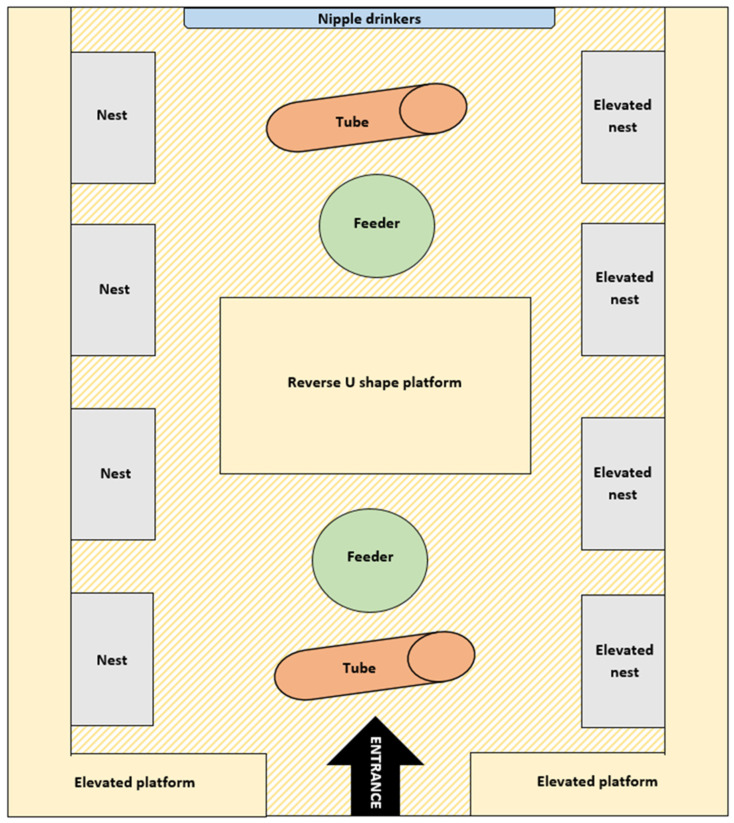
Housing system scheme (housing systems differed between groups by floor type).

The study was piloted at the Czech University of Life Sciences Prague using 72 24-week-old rabbit does: slow-growing does of Mecklenburg rabbits (MC) and commercial hybrid Hyplus, where all housing conditions were simulated. The observations took place from May until July 2021. Rabbits were fed a commercial pelleted diet (Sehnoutek a synové, Czech Republic), water (provided by nipple drinkers), gnawing material (wooden sticks), and hay were available ad libitum. From 10 weeks of age, female rabbits were housed individually (deep litter with access to hay) in the open-air system. At 24 weeks of age, does were randomly assigned to four treatments depending on the floor type—concrete with deep litter (DEEP) or soil (the depth of the soil was ad libitum for rabbits, so the females could dig however they wanted) with deep litter (DIG)—and genotype (MC or Hyplus). The does were divided according to genotype (36 does per genotype) and according to the floor type (DEEP or DIG). Each treatment consisted of three replications of six rabbit does according to genotype and housing system. The does were weighed at the beginning and the end of the experiment (after kindling). The average weight of MC does at the beginning of the experiment was 4561 g ± 169 SE and 4503 g ± 180 SE in Hyplus does. The weight of MC does was 4434 g ± 225 SE and 4208 g ± 165 SE in Hyplus does after kindling on average. After four days of socialisation, a male of the same genotype was released into the group. As specified by [14], the male was present for ten days in each group and then he was removed. After these ten days, all does were detected as gravid by palpation (kits were born during two days). The housing scheme can be seen in Figure 1. Pens had the tops open, and the average size was 3.5 × 2.0 m with a minimum surface area of 1.6 m^2^ per doe. A reverse “U” shape platform (1 × 0.6 m), elevated platform (0.4 m above the ground), and nest boxes (0.3 × 0.4 × 0.3 m) were counted in. The nest boxes were in two positions accessible for the does: either on the ground or in elevated areas. The number of nest boxes was always two more (8 in total) than the number of does in the pen to prevent unwanted aggressive behaviour during the choosing of the nest. In the DIG group, the nest boxes were placed 10 cm below the ground for better stability. Elevated platforms and resting areas were made of wood with no perforation. Moreover, in the central part of the pen, there was a platform with the reverse “U” shape, which also served as a hiding spot. Each housing system was equipped with hiding tubes lying on the floor (1 × 0.25 m). The entire area of the pen (concrete × soil) was completely covered (15 cm depth) with deep straw litter (chopped straw with an average length of 0.1 m), which was removed every week. Housing systems were placed outdoors and protected by netting against intruders.

Growing rabbits, after weaning were visually checked every week for control of their health status until the end of the slaughter (not a part of the present paper).

### 2.2. Behavioural Observation and Analyses

All pens were video recorded with colour infrared cameras (Sikur systems, Frýdek-Místek, Czech Republic). The period of observation had five parts: the initial period of the group formation, when the male was added 1 week before kindling; 14 days after kindling; and 35 days after kindling (contact with growing kits). Videos were recorded twice in 30-min-long intervals (day and night, from 10:00 to 10:30 and 22:00 to 22:30, resp.) during each period. Aggressive behaviour against does (does were marked by different colours) was observed according to [15] in 1-min intervals by the one-zero sampling method (i.e., the behaviour being present at any time during the 1-min interval) for 30-min periods. The sums of values obtained from the reproductive period were counted for each behaviour and doe (marked by spray paint). The video clips were assessed by one trained researcher. Aggressive events were evaluated according to [16] as biting (gripping with the teeth); boxing (hitting with the front paws); chasing (aggressive following of another individual for at least three jumps); carousel-fights (rapid chasing around in one spot with the rear end of the opponent gripped between the teeth); attacking (aggressive running towards another female); and threatening (quick head movement toward another doe). These events were counted together, and the sum of them was considered aggressive behaviour. “Sniffing” was considered as contact between two females who sniff each other without an aggressive ending to the event.

### 2.3. Injury Scoring in Does

The does were individually checked for injuries before their release into the new group (day zero). Then, all does were scored three days after the group formation, three days after the male was added, three days after the male was removed from the group, twice (once a week) before kindling, and six times (once a week) after kindling until the kits were weaned. Each doe was carefully treated by an experienced researcher. Each female was captured and examined, old and healed injuries were excluded after the visual examination (fully or almost fully healed ones), and new ones were counted. The scoring schedule was conducted according to [9], where (0) were no skin injuries; (1) denoted small (<1 cm) superficial skin injuries (scratches), fewer than 6 in total; (2) denoted superficial skin injuries > 1 cm, deeper lesions in the connective tissue or more than five lesions score 1; (3) denoted very deep lesions in the muscle tissue (wounds) or more than five lesions as in score 2. We estimated the does’ injuries by two variables: as a sum of scores; and by counting the number of injuries per doe without scoring. However, since these two variables were highly correlated (*r* = 0.94, *p* < 0.0001), we used the latter variable for the analysis.

### 2.4. Injury Scoring in Kits

Kits were examined from 21 days of age until weaning once a week (three times in total). The sum of injuries in kits was counted for the observed periods. Kits usually did not receive wounds or deep bites (if they do, they were found dead). On their bodies were found in most cases little bites or scratches, so we considered counting the number of injuries on kits as reasonable and did not use the scale.

### 2.5. Postnatal Kits Mortality

Dead kits were recorded during the whole experimental period. Most bitten kits were found dead, so doe attacks were assumed to be the cause of death. Moreover, these kits had visible wounds on their body. In addition, to the total sum of dead kits we also added kits which were found dead in nests during first days after kindling, as the result of a fight between two does in the nest (visible on cameras).

### 2.6. Statistical Analyses

All data were initially tested for normality (Shapiro–Wilk test) and homogeneity of variances. Data validation was approached by calculating information on the validation and/or assurance quality procedure and output. Associations between dependent variables—aggressive behaviour towards other does (aggressive behaviour), does sniffing the other does within the pen (sniffing does), the quantity of skin injuries in does (doe injuries) and injuries in kits, and postnatal kit mortality (characteristics of the dependent variables in Table 1)—and independent variables (listed in Table 1) were tested using multivariate General Linear Mixed Models (GLMM, PROC MIXED in SAS 9.4). All GLMMs were designed for repeated measures (i.e., in SAS, where REPEATED = period; and SUBJECT = identity of the doe nested within the group) with the random effect (in SAS, RANDOM = group nested within housing).

For the dependent variables, aggressive behaviour, sniffing does, and doe injuries, we constructed the models by first entering the interaction between housing and genotype, and then checking the model with the addition of the factors which could also affect the result (Table 1).

We could only measure the dependent variables, injured kits and postnatal kit mortality, during periods 4 and 5. Therefore, for these dependent variables, we removed the repeated measures design and analysed the GLMM with the interaction between genotype and housing and then checked the model with other possible factors from Table 1. Least-squares means (LSMEANs) were computed here and thereafter for each class and, where appropriate, differences between classes were tested by *t*-test. We used a Tukey-Kramer adjustment for multiple comparisons. Associations between the dependent variable and fixed effects were estimated by fitting a random coefficient model using PROC MIXED as described by [17]. We calculated the predicted values of the dependent variables and plotted them against the fixed effects with a regression line. Statistical significance was based on *p* < 0.05.

## 3. Results

In total, we observed 21,600 one-min-long sampling intervals (300 for each doe) during which we registered 439 aggressive events: 143 in MC does, and 296 in Hyplus does. We further registered 847 events when a doe was sniffing another doe, 352 in MC does, and 495 sniffing events in Hyplus does. Overall, we had 212 kits from MC does and 244 kits from Hyplus does. In total, during the experiment, we counted 271 skin injuries on does, 96 on MC does, 175 on Hyplus does, and 554 skin injuries on kits, 177 in MC, and 377 in Hyplus kits. We found 97 dead kits, 25 MC kits, and 72 Hyplus kits.

### 3.1. Number of Attacks among Does

**Figure 2:**
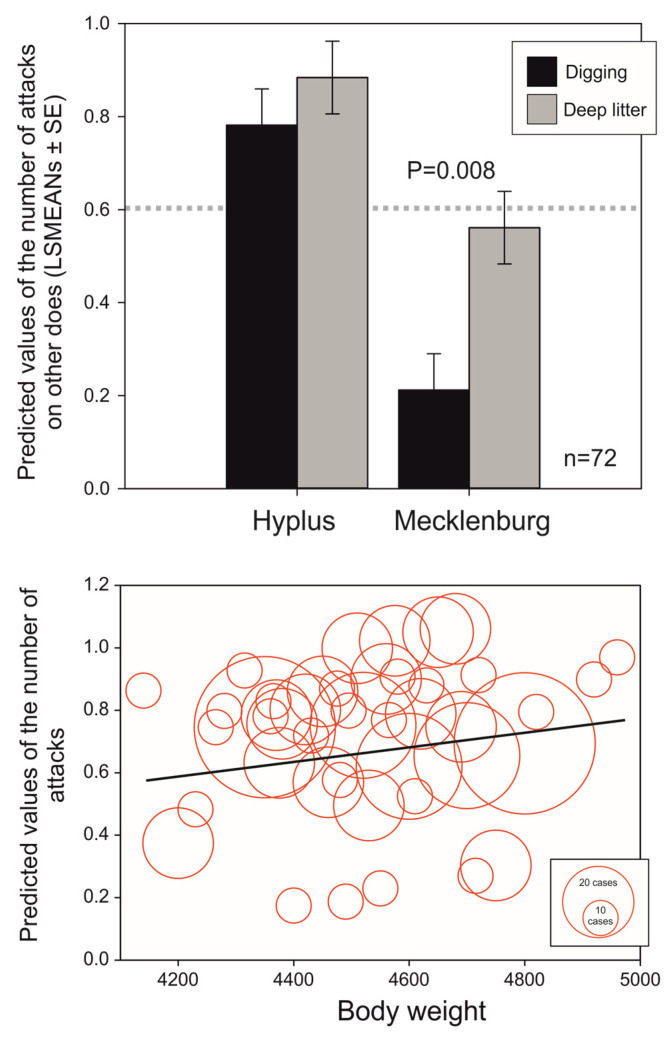
Predicted values of the number of attacks on other does according to housing (**top**) for Hyplus (left column) and Mecklenburg rabbits (right column); LSMEANs ± SE, n = 72 for each column, plotted against the body weight (**bottom**).

In partial agreement with the advanced hypothesis, housing and genotype had a significant effect on the number of attacks among does (Table 2), where MC does from DIG housing performed a smaller number of attacks towards other does compared to MC does from DEEP housing (*p* = 0.0082), as shown in Figure 2. There were also significant differences when comparing does from the same housing treatment but different genotypes. MC does from DIG housing had fewer attacks compared to Hyplus does from DIG housing (*p* = 0.0002), and MC does from DEEP housing had less attacks compared to Hyplus does from DEEP housing (*p* = 0.0128). However, there was no significant difference in the number of attacks between Hyplus does from DIG and Hyplus does from DEEP housing.

In addition, body weight had a significant effect on the number of attacks among does (Table 2), with heavier females attacking significantly more often than lighter females (Figure 2).

### 3.2. Doe Sniffing the Other Does

Differences in housing and genotype did not reach statistical significance for sniffing among the does within the pen (Table 2).

### 3.3. Number of Skin Injuries of Does

**Figure 3:**
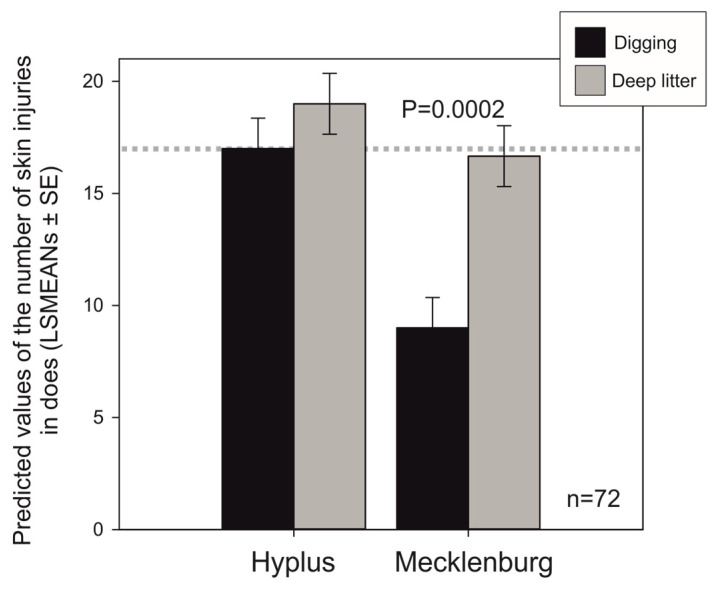
Predicted values of the number of skin injuries in does according to housing for Hyplus (left column) and Mecklenburg rabbits (right column); LSMEANs ± SE, n = 72 for each column.

The number of skin injuries and the severity of injuries were strongly correlated (*r* = 0.942). Reduced aggression was reflected in a lower number of injuries in does (Table 2, Figure 3), where MC does from DIG housing had fewer skin injuries compared to MC does from DEEP housing (*p* = 0.0092). There were also significant differences when comparing does from the DIG housing treatment but different genotypes. MC does from DIG housing had fewer injuries than Hyplus does from DIG housing (*p* < 0.0001). However, there were no significant differences in the number of injuries in MC does from DEEP housing compared to Hyplus does from DEEP housing and no differences in the number injuries between Hyplus does from DIG housing and Hyplus does from DEEP housing. Body weight also had no effect on the number of skin injuries.

### 3.4. Number of Injuries in Kits

**Figure 4:**
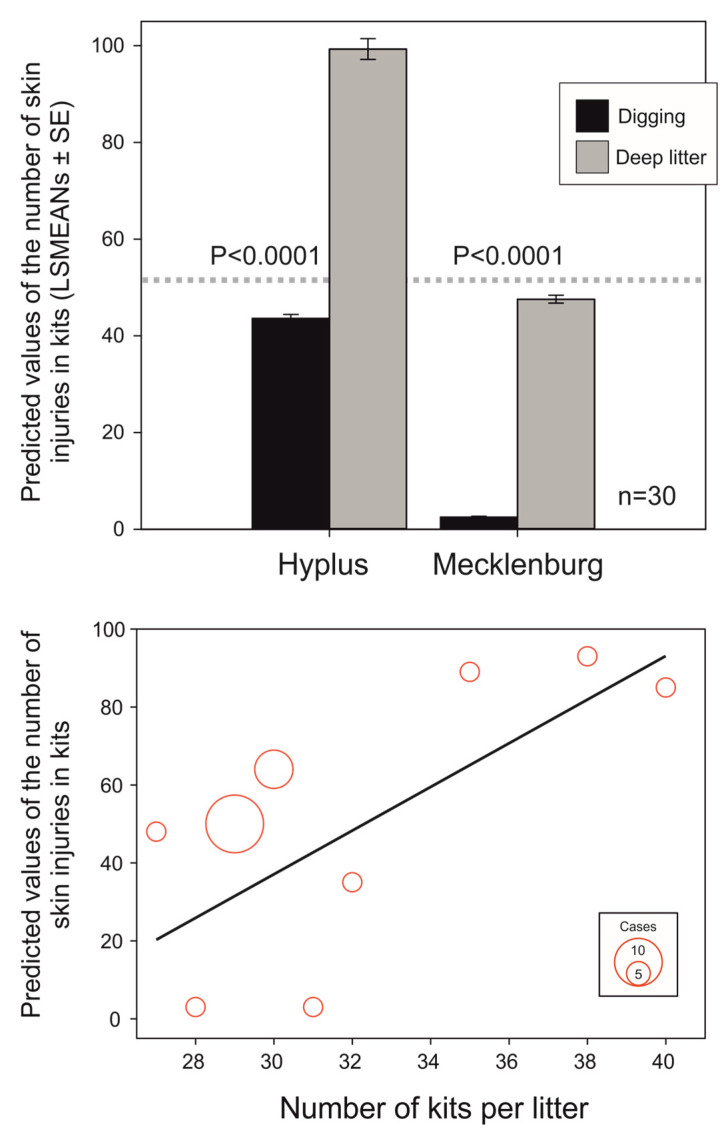
Predicted values of the number of skin injuries in kits according to housing (**top**) for Hyplus (left column) and Mecklenburg rabbits (right column); LSMEANs ± SE, n = 30 for each column, plotted against the number of kits per litter (**bottom**).

The housing treatment in interaction with the genotype had a significant effect on the injuries of kits (Table 2, Figure 4), where MC kits from DIG housing had fewer injuries than MC kits from DEEP housing (*p* < 0.0001) and Hyplus kits from DIG housing had fewer injuries than Hyplus kits form DEEP housing (*p* < 0.0001). Furthermore, the number of injuries of kits was also significantly affected by the size of the litter (Table 2); the more kits were in the litter, the more injuries the kits had (Figure 4).

### 3.5. Postnatal Kit Mortality

**Figure 5:**
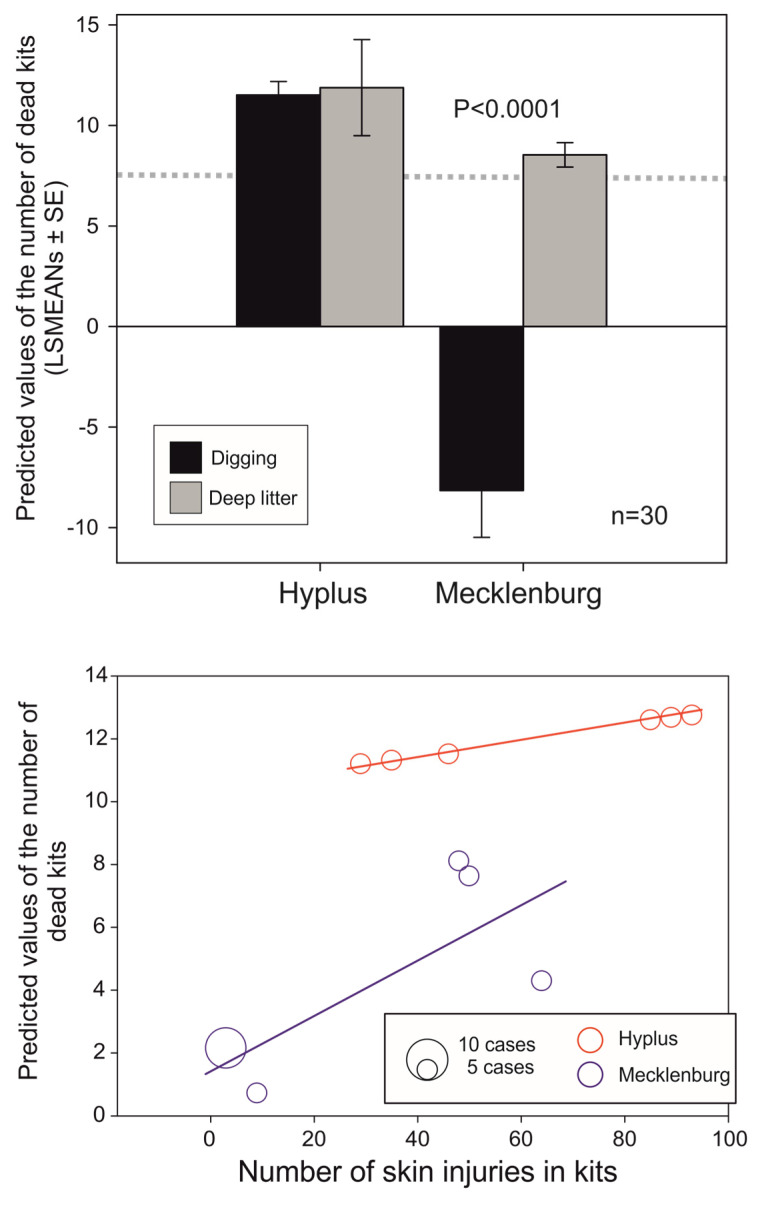
Predicted values of the number of dead kits according to housing (**top**) for Hyplus (left column) and Mecklenburg rabbits (right column); LSMEANs ± SE, n = 30 for each column, plotted against the number of skin injuries in kits (**bottom**) for Hyplus (red) and Mecklenburg rabbits (blue).

Housing and genotype also had a significant effect on postnatal kit mortality (genotype nested within housing *F*_3,54_ = 43.94, *p* < 0.0001). Kit mortality was lower in MC kits in DIG housing than in MC kits in DEEP housing (*p* < 0.0001) as seen in Figure 5. There were also significant differences when comparing the mortality of kits from the DIG housing treatment with different genotypes. MC kits from DIG housing had lower mortality than Hyplus kits from DIG housing (*p* < 0.0001). However, there were no significant differences in the mortality of MC kits from DEEP housing compared to Hyplus kits from DEEP housing. The mortality rate was also no different for Hyplus kits from DIG housing compared to Hyplus kits from DEEP housing. The number of injuries had a significant effect on mortality (injuries nested within genotype *F*_2,54_ = 9.28, *p* = 0.0003). In both genotypes, the higher the number of kits injured, the higher the kit mortality (Figure 5).

## 4. Discussion

The results of the present study showed that the housing system and genotype had a significant effect on the aggressive behaviour of rabbit does. Generally, when the does were housed in groups (continuous or part-time), they manifested a high frequency of aggressive events before the hierarchy of the group was established [18,19,20]. This trend was also observed in the present study, where both genotypes showed a high level of aggressive behaviour in the first observed period. Several studies tried to solve the problem of aggression with different regrouping schedules [21] or multiple strategies, when platforms, plastic pipes, hiding places, straws, dark corridors, or sprayed odours were tested, and the results were rather unconvincing, as reviewed by [7]. These findings were used in our study, where the deep litter systems were equipped with appropriate (previously tested) elements (such as hiding pipes, gnawing sticks, and shelters), and the digging systems were enriched with the same elements and the possibility of creating a rabbits’ warrens under the ground and doing related activities.

In our study, the commercial Hyplus was more aggressive in general than the MC genotype. However, their inter-individual social behaviour, reflected in sniffing, did not differ. It is likely that Hyplus does are more aggressive than MC does because they are selected primarily for productive parameters [22], regardless of behaviour. Contrarily, on small farms or at breeders (these breeders typically use the original or local breeds on their farms), it is very common to eliminate aggressive rabbits from breeding, as the rabbits are frequently used as pets [23]. Another possible explanation for our results could be hidden in the hypothesis which postulates that some individuals possess genetic variants enhancing their vulnerability to environmental adversity. This was reported by [24] in humans, where the longitudinal data of several hundred people were used, resulting in relatively common variants of the dopamine receptor gene and the serotonin transporter gene interacting with social conditions to predict aggression in a manner consonant with the differential susceptibility perspective. The authors reported that in case of adverse environmental conditions, the individuals with these genetic variants manifested more aggression than other genotypes. Additionally, the effect of genotype on aggressive behaviour was previously confirmed by [25], who observed differences in expressing this behaviour among various mouse strains. This is also well-known in chickens, where white-feathered birds are more aggressive than red ones [26], which is generally correlated with some candidate genes [27]. However, these studies considered that genetics could be the key determinant of aggressive behaviour; studies related to humans are in this way more detailed. Moreover, we found a higher number of attacks directed from heavier to lighter does. The information about the effect of body weight on aggressive behaviour is lacking in the scientific literature. Only [28] reported no correlation between body weight and rank order in growing rabbits from 4 to 12 weeks of age.

Aggression is not reflected just in hurting other does, but also in injured or even killed kits. Ref. [29] reported that wild rabbit does tolerate their kits but demonstrate aggression against other offspring. In addition, aggression can exceed into infanticide due to the limited number of burrows. These events naturally lead to increased kit mortality and therefore economic losses. In the present paper, a higher incidence of mortality was observed in the Hyplus genotype, which was more aggressive compared to MC does, as was previously mentioned.

The housing system influenced aggression in the less aggressive MC genotype only. Hyplus does maintain aggression at a high level during the whole reproductive period. It has to be emphasised, however, that the does’ injuries had never been severe enough to lead to inflammatory conditions. Still, we recorded more injured does and kits in DEEP systems compared to DIG systems. Surprisingly, the injuries led to mortality only among the MC genotype. The greater part of dead kits among the Hyplus genotype occurred during the first days after leaving of the nest. The aggression is mostly noted at the start of the reproductive season and after parturition, when rabbit does are intolerant of each other [30]. Problems with aggression are therefore reported with respect to both strategies of housing rabbit does. Ref. [6] tested four individual modules and a common area, where the rabbit does were mixed after 18 days after kindling, resulting in 50% injured does. Furthermore, [31] observed groups of five does in a part-time system with results showing 34% injured does four days after group formation and 53% injured does at litter weaning. In addition, during lactation, kits were the subject of aggressive attacks from other does. These attacks can be then the cause of death as assumed in [32], who observed a significant effect of grouping rabbit does at different times post-partum. In our study, the MC genotype housed in DIG systems seemed to be more compatible than Hyplus. Hyplus may manifest more aggressive behaviour and related injuries, or deaths could occur due to their “higher distance” from wild rabbits as these lines are bred specifically for high production. These findings could be explained by the study of [33], who compared domestic and wild rabbits and found a higher number of aggressive attacks among wild rabbits than their domestic counterparts. Based on the results, it seems that the genetic selection of reproductive traits could also affect the maternal behaviour of rabbit does, which, in selected strain, could increase aggressive behaviour in protecting the nest and the litter. In the present study, the MC does are calmer; they do not look for fights. On the contrary, Hyplus fights more for hierarchy and may need a bigger space for their nests to eliminate attacks from other does or kits.

## 5. Conclusions

The results show that the choice of genotype and housing system is crucial with respect to aggressive interactions among rabbit does. Aggression results in injuries among rabbit does and their kits. Fights among does can sometimes occur in the nest and new-born kits will subsequently die. Kits that are seriously injured by other rabbit does later die. The MC genotype, as the original medium-heavy breed, is less aggressive than the commercial hybrid we tested and can be recommended for smallholder breeding conditions. For these farmers, it is also important to choose a suitable housing system; according to our results, the possibility of digging should not be missing, and housing systems should accommodate the building of nests of greater complexity. In addition, although we did not observe any health problems in does or their kits, the potential risk of coccidiosis infection is present when using deep litter instead of a slatted or wire floor. It would be interesting to test the genotypes of rabbits typically used for meat production on small farms in future research. Moreover, other studies should focus on the effect of genetic selection on the maternal behaviour of rabbit does.

## Figures and Tables

**Table 1:** Countable and categorical variables available of moderating rabbit does aggressiveness by housing conditions and genotype.

Countable Variables	Mean	SE ^1^	Min	Max	Unit
Aggressive behaviour	0.61	0.04	0	7	Number of cases
Sniffing does	1.18	0.07	0	15	Number of events
Doe injuries	112.92	2.59	0	330	Number of injuries
Kit´s injuries	46.17	4.01	3	93	Number of injuries
Kit natality	38	3.98	30	45	Number of kits born per group
Postnatal kit mortality	8.08	0.61	1	14	Number of dead kits per group
Doe’s body weight	4531	6.52	4140	4960	g
Categorical variables		Levels			Description
Housing		2			DEEP ^2^/DIG ^3^
Genotype		2			Hyplus/MC ^4^

^1^ SE. standard error; ^2^ DEEP, deep litter housing; ^3^ DIG, digging housing system; ^4^ MC, Mecklenburg rabbits.

**Table 2:** GLMM results (fixed effects, degrees of freedom, *F*-Value, and probability) of moderating rabbit does aggressiveness by housing conditions, and genotype.

Fixed Effect	Num DF	Den DF	*F*-Value	*p*-Value
Dependent variable: aggressive behaviour				
Housing*genotype	3	12	14.34	0.0003
Body weight	1	62.6	4.93	0.0301
Dependent variable: sniffing				
Housing*genotype	3	12	2.67	0.0946
Dependent variable: injuries in does				
Housing*genotype	3	68	20.40	<0.0001
Dependent variable: injuries in kits				
Housing*genotype	3	1	4.59	<0.0001
Litter size	1	60	8.71	0.0045
Dependent variable: postnatal kit mortality				
Housing*genotype	3	54	43.94	<0.0001
Number of injuries in kits (genotype)	2	54	9.28	0.0003

*, It is a sign for interaction.

## Data Availability

The data are available upon reasonable request.
